# Adherence with blood pressure monitoring wearable device among the elderly with hypertension: The case of rural China

**DOI:** 10.1002/brb3.1599

**Published:** 2020-05-09

**Authors:** Yuting Zhang, Yuan Fang, Yi Xu, Peng Xiong, Jingyi Zhang, Jinru Yang, Li Ran, Xiaodong Tan

**Affiliations:** ^1^ Department of Occupational and Environmental Health School of Health Sciences Wuhan University Wuhan China; ^2^ Department of Nursing The Second Affiliated Hospital of Bengbu Medical College Bengbu China; ^3^ Department of Preventive Medicine and Public Health School of Medicine Jinan University Guangzhou China; ^4^ College of Clinical Medicine Wuhan University of Science and Technology Wuhan China

**Keywords:** adherence, blood pressure monitor, elderly, hypertension, mHealth, rural China, wearable device

## Abstract

**Introduction:**

Wearable blood pressure (BP) monitor devices are increasingly adopted owing to the promotion of hypertension management program. However, little is known about the adherence and its associated factors in older adults (OAs) with hypertension.

**Methods:**

The aim of this study was to determine factors associated with adherence to wearable BP monitor. In total, 212 OAs with hypertension in a remote rural area in China were asked to wear a BP monitor over 1‐month period. The following information on associated factors for adherence was collected, including demographic characteristics, cardiovascular health measurements, technology fluency, the Compliance of Hypertensive Patients' Scale, and the Health‐related Quality of Life Survey. As a result, the mean age of the 212 hypertension participants was 71.32 years (*SD* = 6.81).

**Results:**

During the 30‐day survey, 50.94% of the participants with daily recoded BP wristband data were assigned as “BP device users.” The binary logistic regression model revealed that lower lifestyle compliance, lower medication compliance and higher total hypertension compliance were significant predictors for adherence. However, there were no differences concerning cardiovascular health factors, technology fluency, and health‐related quality of life between device users and nonusers.

**Conclusion:**

Individuals reporting a higher level of total compliance in hypertension management were more likely to wear a BP monitor device among OAs with hypertension. In addition, further research is needed to determine how wearable mHealth technology can be used to develop better hypertension self‐management education programs for this population.

## Graphical abstract

Blood pressure (BP) monitoring wearable devices are increasingly being adopted within hypertension management program. However, little is known about the adherence and its associated factors of older adults (OAs) with hypertension. We found that individuals reporting a total higher level of compliance in hypertension management were more likely to wear a BP monitoring wearable device among OAs with hypertension.

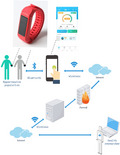

## 1 INTRODUCTION

Hypertension, a primary contributor to cardiovascular disease (CVD), is more prevalent among older adults (OAs) than young adults (Chen, Hu, Mccoy, Letvak, & Ivanov, 2018). According to the American Heart Association (Benjamin et al., 2018), the prevalence of hypertension was estimated to be 34.0% among US adults aged over 20 years, while 67.2% in those aged over 65 years. Meta‐analytic evidence (Ettehad et al., 2016) has demonstrated that a 10 mm Hg reduction in blood pressure (BP) could hopefully lead to a 41% reduction in CVD and a 37% reduction in heart failure. Consistently, a SPRINT clinical trial (Lu et al., 2017; Wright et al., 2016) has reported that further reductions could lead to significantly better health outcomes. However, the awareness, treatment, and control of hypertension among Chinese adults aged 35–75 years are all suboptimal (Lu et al., 2017).

Mobile health (mHealth), defined as medical practice supported by mobile devices, including smartphones, wearable devices, and other patient monitor devices, has shed novel light on health management (Ryu, 2012). mHealth interventions offer opportunities to enhance both BP screening and ongoing management in a potentially cost‐effective pattern (Staffileno, Tangney, Fogg, & Darmoc, 2015). Several studies (Kaambwa et al., 2013; Margolis et al., 2013; Uhlig, Patel, Ip, Kitsios, & Balk, 2013) have shown that interventions by combining with intensive support, such as patient education, social support, and group behavioral treatment with patient self‐monitoring BP devices are likely to effectively control, thereby decreasing BP. However, the reported efficacy and effectiveness of healthcare interventions are challenged by multiple factors, particularly how these mHealth devices are essential to acceptable and effective adherence for remote delivery (Morrison, Lucy, John, & Susan, 2012). To the best of our knowledge, studies have rarely focused on the influencing factors with the adherence with wearable BP monitor device in a remote rural area in China.

To date, several epidemiologic studies have assessed the compliant wearable device users (Glynn et al., 2015; Mair et al., 2012; Murray et al., 2010). Yet, little information is available about the specific features contributing to adherent device users in the community‐based elderly population. To this end, the present study was designed to evaluate the differences in sociodemographic characteristics, cardiovascular health factors, technological fluency, hypertension compliance, and health‐related quality of life between device users and nonusers in the elderly. The ultimate goal of our study was to provide potential future directions for the mHealth technology in this field.

## 2 METHODS

### 2.1 Study population

**Figure 1:**
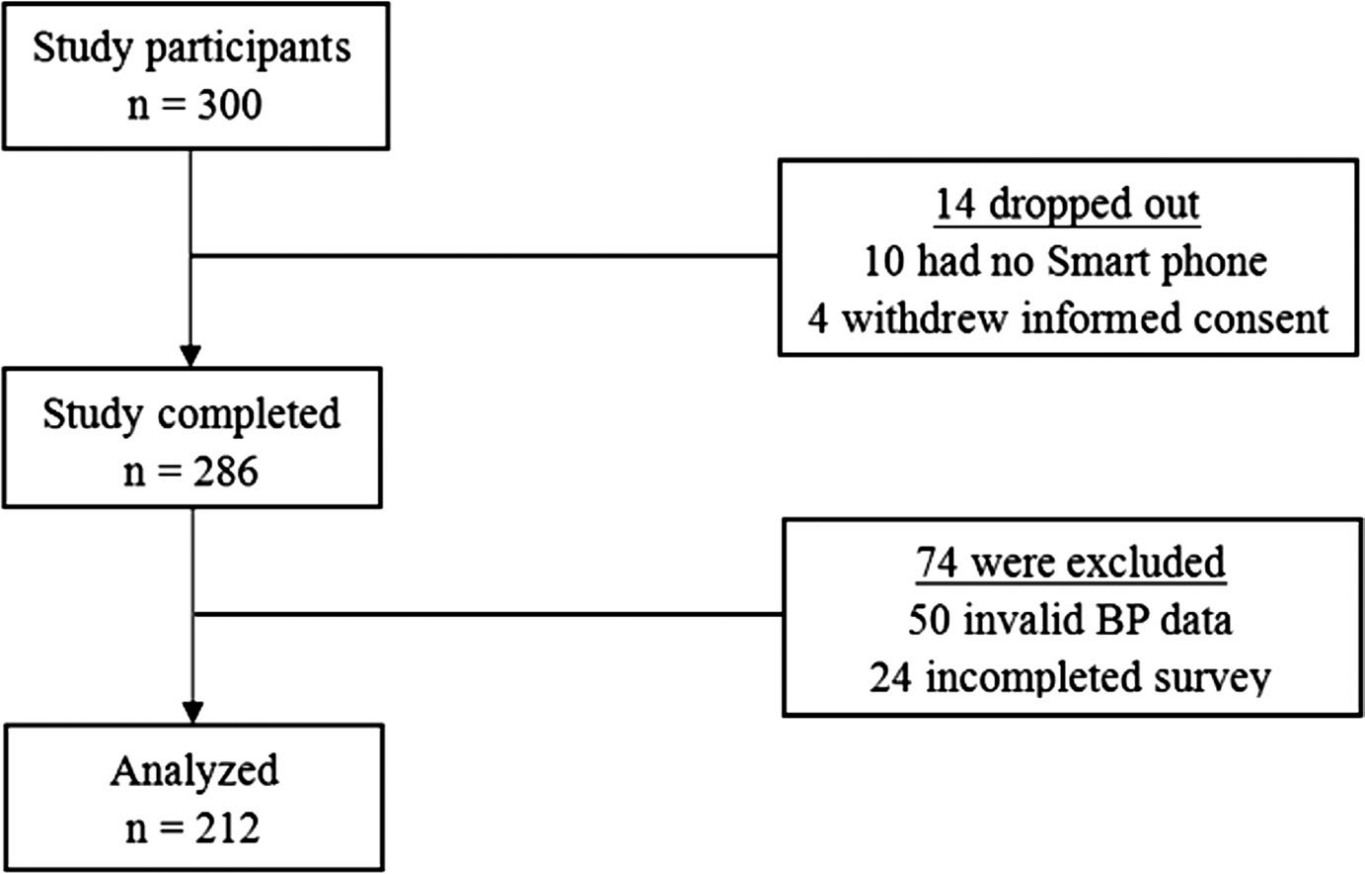
Disposition of participants

Individuals with confirmed hypertension were recruited for a self‐monitoring intervention program for hypertension control in a remote mountainous district of Hubei province from April 2018 to July 2018 (the disposition of participants was shown in Figure 1). The inclusion criteria were shown as follows: (a) living in the Xuanen area; (b) aged over 60 years; (c) systolic BP ≥140 mm Hg and/or diastolic BP ≥90 mm Hg or a history of antihypertensive medication; and (4) communication proficiency to carry out study tasks. Exclusion criteria were as follows: (a) cognitive dysfunction; (b) without smartphones to use the device; and (c) hypertension emergency or other urgency.

Participants completed the study in the following orders: (a) registration and signing written informed consent; (b) measurement of BP, height, weight, waistline, and hipline; (c) survey instrument completion; (d) mHealth device training; and (e) debriefing. The collection of BP data continued throughout the 30‐day period.

The study was approved by the institutional review boards of Wuhan University, and written informed consent was obtained from all participants.

### 2.2 BP data collection

**Figure 2:**
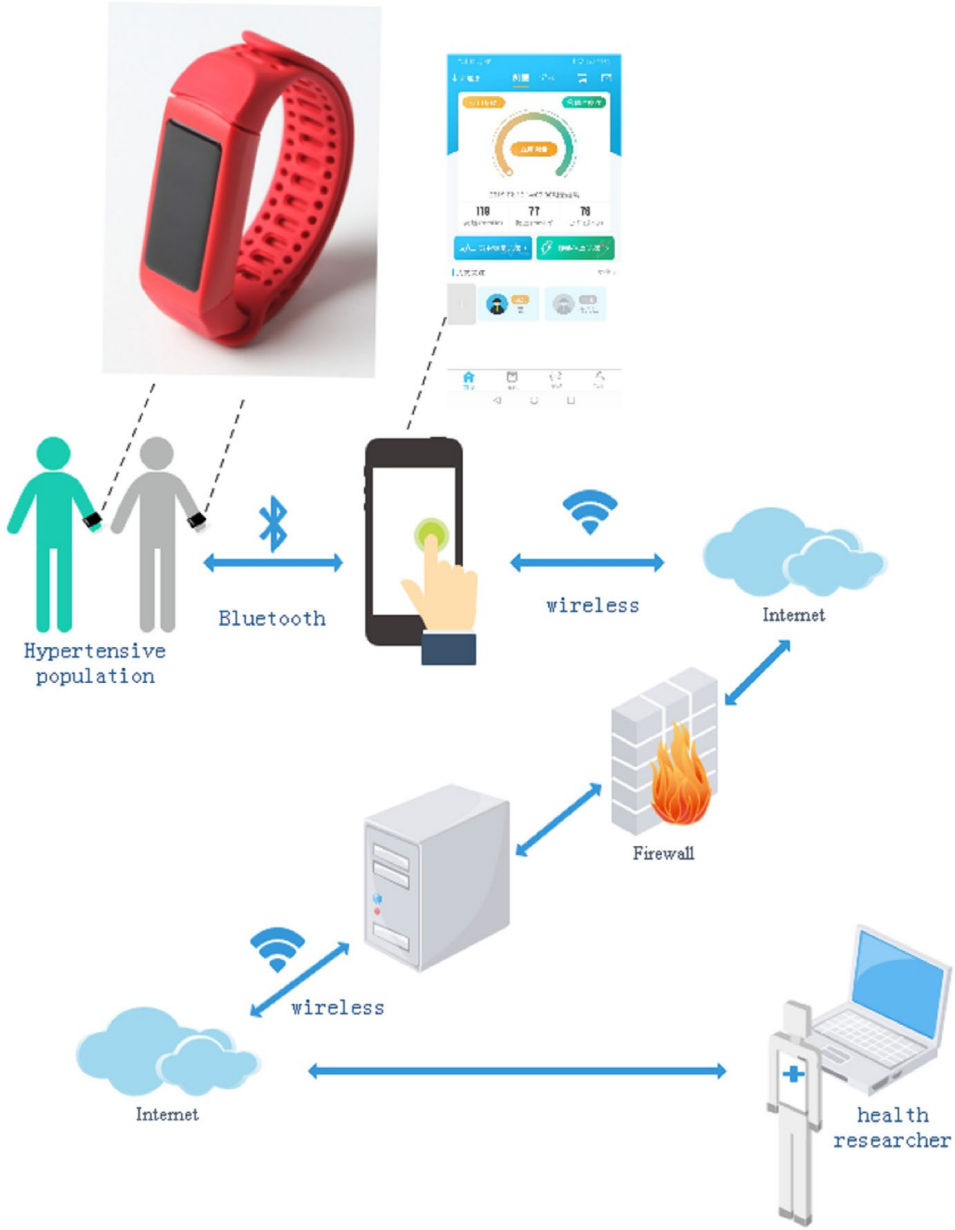
Secure data collection system for blood pressure monitor data

A 12‐hr ambulatory BP monitor was used to assess the BP of participants. The device was pilot‐tested from December 2017 to February 2018 among a similar elderly population in Wuhan, China (*N* = 98). The accuracy and reliability of the device were validated. Based on previous studies, the monitor was designed to automatically record BP at 1‐hr interval during awake hours (Celler, Argha, Varnfield, & Jayasena, 2018). The BP monitoring system also included a secure online account to track CVD‐related health factors (Figure 2). A hub was installed in an accessible location at each community. The wristband device could automatically upload the recorded BP to central hub location through a smartphone application daily during the 1‐month study. Pressing the device button would prompt the smartphone to display BP results. The system would terminate the transmission of data if there was no Internet connection. Upon receipt of the ambulatory BP monitor, participants were trained on the usage of the wristband and provided with a written instruction manual. More details of the research process were reported elsewhere (Zhang, Tan, Si, & Huang, 2019; Zhang, Xu, et al., 2019; Zhang, Yang, et al., 2019). Each community had a “super user,” a participant volunteer who helped community participants with hub issues and communicated with investigators. The uploaded BP data were collected and the device usage of participants was monitored by investigators during the 30‐day study period.

All BP wearable devices were provided for free.

### 2.3 Measures

#### 2.3.1 Definition of adherence

Adherence to BP monitor was defined as daily recording of BP data during the 30‐day assessment period. “BP device user” (coded “1”) referred to participants with daily uploaded BP results over the study period. While participants without daily recorded BP data were noted as “BP device nonuser” (coded “0”).

#### 2.3.2 Computer–email–web fluency

Utilization of mobile phone and Internet technology was evaluated using the adapted Computer–Email–Web (CEW) Fluency Scale developed by Bunz (2004). The CEW is one of the most widely used scales to measure levels of technology fluency in OAs (Bunz, Curry, & Voon, 2007). The generally adapted CEW is a 17‐item scale, all of which are rated on a 5‐point Likert scale ranging from 1 (no fluency) to 5 (high fluency) based on three subscales (computer skills, email skills, and web skills). Higher scores indicated higher levels of fluency. In this study, the Cronbach's alpha of the CEW was 0.985.

#### 2.3.3 Hypertension compliance

Hypertension compliance was measured using the Compliance of Hypertensive Patients' scale (CHPS) developed by Kyng and Lahdenper (2010). CHPS is one of the most commonly used instruments to evaluate hypertension compliance in OAs (Lahdenperä, Wright, & Kyngäs, 2003). To be specific, CHPS, a 15‐item scale, could be used to assess the level of compliance in the following six aspects: Intention (4 items), Lifestyle (3 items), Attitude (3 items), Responsibility (2 items), Smoking and drinking (2 items), and Medication use (1 item). Higher scores suggested higher levels of compliance. The Cronbach's alpha of the CHPS was 0.859 in this study.

#### 2.3.4 Health‐related quality of life

Health‐related quality of life (HRQOL) was determined using the 12‐Item Short‐Form Health Survey (SF‐12), which was originally developed as a short alternative to the SF‐36. The SF‐12, a widely used generic questionnaire, has been widely validated to measure HRQOL in OAs (Bentur & King, 2010; Jakobsson, 2007). It is measured by two summarized components: a physical component score (PCS) and a mental component score (MCS). Both PCS and MCS scores range from 0 to 100 according to the scoring program described by Ware, Kosinski, and Keller (1996). Higher scores of each component indicated higher levels of life quality. In this study, the Cronbach's alpha of the SF‐12 was 0.828.

### 2.4 Data analysis

SPSS software version 21.0 was used for statistical analysis. Descriptive baseline sociodemographic characteristics of the participants were presented as percentages. Independent *t* test (for continuous variables) and chi‐squared test (for categorical variables) were utilized for comparison between user and nonuser groups. Afterward, a binary logistic regression analysis was constructed to determine the significant variables associated with the adherence to device use. The variables selected for regression model were based on all variables from the bivariate analysis with *p* ≤ .05. A two‐sided *p* ≤ .05 was considered as statistically significant.

## 3 RESULTS

The baseline characteristics of the users and nonusers were shown in Table 1. In total, 70.67% participants completed all survey and measurements at baseline. Of the 212 subjects, 110 (51.89%) were female, with the mean age of 71.32 years (standard deviation: 6.81, range: 60–98). During the 30‐day study period, 50.94% of the participants uploaded BP wristband data to central hub location at least once daily. The family monthly income (*p* = .040) and number of concomitant diseases (*p* = .043) were significantly different between users and nonusers.

**Table 1:** Sociodemographic characteristics of participants (*N* = 212)

Variable	Total	Users (*n* = 108)	Nonusers (*n* = 104)	*χ^2^*	*p*
Gender					
Male	102 (48.11)	51 (47.22)	51 (49.04)	0.070	.891
Female	110 (51.89)	57 (52.78)	53 (50.96)		
Age (years)					
60–69	102 (48. 11)	56 (51.85)	46 (44.23)	2.689	.277
70–79	80 (37.74)	35 (32.41)	45 (43.27)		
≥80	30 (14.15)	17 (15.74)	13 (12.50)		
Ethnic group					
Han	80 (37.74)	42 (38.89)	38 (36.54)	2.739	.614
Tujia	73 (34.43)	37 (34.26)	36 (34.62)		
Miao	30 (14.15)	14 (12.96)	16 (15.38)		
Dong	24 (11.32)	14 (12.96)	10 (9.62)		
Other	5 (2.36)	1 (0.93)	4 (3.85)		
Marital status					
Married	174 (82.08)	85 (78.70)	89 (85.58)	1.701	.213
Single	38 (17.92)	23 (21.30)	15 (14.42)		
Years of schooling					
≤6	141 (66.51)	70 (64.81)	71 (68.27)	1.963	.587
7–9	31 (14.62)	19 (17.59)	12 (11.54)		
10–12	23 (10.85)	10 (9.26)	13 (12.50)		
≥13	17 (8.02)	9 (8.33)	8 (7.69)		
Primary occupation (before age 60)					
Governmental personnel	51 (24.06)	22 (20.37)	29 (27.88)	4.820	.422
Service worker/industrial worker	24 (11.32)	12 (11.11)	12 (11.54)		
Self‐employed	9 (4.25)	3 (2.78)	6 (5.77)		
Farmer	113 (53.30)	64 (59.26)	49 (47.12)		
Medical staff	1 (0.47)	0	1 (0.96)		
Unemployed	14 (6.60)	7 (6.48)	7 (6.73)		
Years of hypertension					
<1	12 (5.66)	7 (6.48)	5 (4.81)	3.907	.425
1–3	29 (13.68)	14 (12.96)	15 (14.42)		
4–5	36 (16.98)	19 (17.59)	17 (16.35)		
6–10	53 (25.00)	32 (29.63)	21 (20.19)		
>10	82 (38.68)	36 (33.33)	46 (44.23)		
Family monthly income (Yuan)					
≤1,000	33 (15.57)	20 (18.52)	13 (12.50)	11.520	.040
1,001–3,000	74 (34.91)	36 (33.33)	38 (36.54)		
3,001–5,000	53 (25.00)	34 (31.48)	19 (18.27)		
5,001–8,000	21 (9.91)	7 (6.48)	14 (13.46)		
8,001–10,000	12 (5.66)	3 (2.78)	9 (8.65)		
≥10,001	19 (8.96)	8 (7.41)	11 (10.58)		
Number of concomitant diseases					
0	42 (19.81)	28 (25.93)	14 (13.46)	8.150	.043
1	111 (52.36)	48 (44.44)	63 (60.58)		
2	35 (16.51)	17 (15.74)	18 (17.31)		
≥3	24 (11.32)	15 (13.89)	9 (8.65)		

The cardiovascular‐related health factors for both users and nonusers were shown in Table 2. To be specific, body mass index (BMI; *p* = .008) and waist circumference (*p* = .005) were significantly different between users and nonusers, while no significant differences were identified on systolic blood pressure (SBP), diastolic blood pressure (DBP), or hip circumferences between the two groups.

**Table 2:** Cardiovascular health factors of participants (*N* = 212)

Variable	Users (*n* = 108)		Nonusers (*n* = 104)		*t*	*p*
	Mean	*SD*	Mean	*SD*		
BMI (kg/m^2^)	24.07	3.17	25.34	3.67	2.698	.008
SBP (mm Hg)	147.02	16.86	151.05	22.34	1.476	.142
DBP (mm Hg)	87.10	12.07	88.43	14.22	0.736	.463
Waist circumference (cm)	87.97	10.09	92.13	11.36	2.824	.005
Hip circumference (cm)	95.15	9.09	97.33	7.41	1.909	.058

As depicted in Table 3, all participants reported some level of technology fluency other than “Not at all” on technology fluency for all three subscales. No differences were identified in computer skills (*p* = .439), email skills (*p* = .453), web navigation skills (*p* = .540), or the cumulative score (*p* = .453) between the two groups.

**Table 3:** Technology fluency of participants (*N* = 212)

Variable	Users (*n* = 108)		Nonusers (*n* = 104)		*t*	*p*
	Mean	*SD*	Mean	*SD*		
Computer skills	7.02	3.29	7.41	4.09	0.776	.439
Email skills	6.74	3.48	7.13	3.96	0.752	.453
Web navigation skills	5.94	3.10	6.22	3.67	0.613	.540
Cumulative score	19.69	9.17	20.76	11.37	0.752	.453

As shown in Table 4, intention (*p* < .001), lifestyle (*p* < .001), attitude (*p* = .001), medication use (*p* = .003), and the total hypertension compliance score (*p* < .001) were statistically significant in hypertension compliance. However, there was no significant difference on responsibility (*p* = .962) or smoking and drinking (*p* = .753) between the two groups.

**Table 4:** Hypertension compliance of participants (*N* = 212)

Variable	Users (*n* = 108)		Nonusers (*n* = 104)		*t*	*p*
	Mean	*SD*	Mean	*SD*		
Intention	11.81	3.09	13.29	2.50	3.819	<.001
Lifestyle	5.69	2.64	7.57	2.19	5.660	<.001
Attitude	9.67	2.12	10.60	1.71	3.518	.001
Responsibility	7.61	0.77	7.61	0.84	−0.048	.962
Smoking and drinking	7.27	1.20	7.21	1.43	−0.316	.753
Medication use	3.04	1.13	3.48	0.99	3.048	.003
Total compliance score	45.08	7.55	49.75	6.63	4.776	<.001

As shown in Table 5, both PCS (*p* = .597) and MCS (*p* = .532) scores were not significantly different.

**Table 5:** Health‐related quality of life of participants (*N* = 212)

Variable	Users (*n* = 108)		Nonusers (*n* = 104)		*t*	*p*
	Mean	*SD*	Mean	*SD*		
Physical health (PCS)	38.07	10.79	38.85	10.52	0.530	.597
Mental health (MCS)	51.22	9.17	50.42	9.49	−0.625	.532

The results from binary logistic regression models were summarized in Table 6. The higher levels of adherence to BP monitor were significantly found in those with lower lifestyle compliance, lower medication compliance and higher total hypertension compliance, while family monthly income, number of concomitant disease(s), BMI, waist circumference, intention, or attitude failed to account for significant variance in this model.

**Table 6:** Binary logistic regression predicting patient's device adherence (*N* = 212)

Variable	*B*	*SE*	Wals	Odds ratio	95% CI	*p*
Family monthly income (Yuan)	−0.024	0.109	0.047	0.977	0.789–1.210	.828
Number of concomitant disease(s)	−0.188	0.181	1.075	0.829	0.581–1.182	.300
BMI	−0.068	0.051	1.791	0.934	0.845–1.032	.181
Waist circumference (cm)	0.004	0.014	0.064	1.004	0.976–1.032	.800
Intention	−0.248	0.130	3.610	0.780	0.604–1.008	.057
Lifestyle	−0.543	0.113	23.055	0.581	0.465–0.725	.000
Attitude	−0.136	0.174	0.610	0.873	0.621–1.228	.435
Medication use	−0.393	0.192	4.203	0.675	0.464–0.983	.040
Total compliance score	0.233	0.076	9.454	1.262	1.088–1.464	.002

Note

Device adherence coding: BP device user = 1 and BP device nonuser = 0.

## 4 DISCUSSION

The study was aimed to explore the differences in sociodemographic characteristics, cardiovascular‐related health factors, technology fluency, hypertension compliance, and health‐related quality of life between users and nonusers. Our results revealed that individuals reporting lower compliance with healthy lifestyle and medication use, but higher total hypertension compliance, were more likely to use mHealth BP monitor over the 30‐day period. In addition, half of the participants (51.89%) were able to wear the device throughout the entire study duration in our study.

To our knowledge, it is one of the first studies to explore user adherence of a BP monitor among OAs with hypertension in a resource‐limited area. As a result, our findings revealed that hypertension compliance was an essential component in the adherence to BP monitor. Consistently, Drevenhorn, Kjellgren, and Bengtson (2010) has indicated that “health lifestyle” is not only related to medication adherence behavior, but also associated with self‐management behaviors including diet, exercise, and weight control. However, our results also suggested that OAs with lower “health life” compliance and medication were more likely to adopt the BP monitor. It might be due to the fact that the study population generally lived with their children and took care of their grandchildren or performed other important tasks for their families. OAs shared their concerns by communicating with family members to cope with the challenges of BP management under the guidance of family members. In a previous study by Chang, Lu, Yang, and Pin (2016), social support exerts a positive effect on users' engagement and adherence with activity trackers. Furthermore, Piette et al. (2015) have asserted that incorporation of caregivers into a mHealth patient monitoring intervention leads to improved medication adherence and improved quality of life among patients with depression. Our findings also underscored the importance of improving hypertension compliance when designing an effective intervention for hypertension control. Therefore, further research is warranted to identify the role of hypertension compliance in device adoption in population with more diverse backgrounds.

In addition, we also found technology fluency as an insignificant factor in the regression model on device adherence prediction, which was consistent with the study by Yingling et al. (2017) on African American population, indicating that lower technology did not appear to impede adoption of mHealth. This relationship attributing similar access to wearable device for both users and nonusers is consistent with the growing widespread mHealth device adoption ownership (Coughlin & Smith, 2016). However, in other trials (Angladamartínez, Roviraillamola, Martinconde, Sotocamomblona, & Codinajané, 2016; Karhula et al., 2015), difficulty in operating technology could result in a lack of device adherence. Other research groups also tried to identify specific age‐related barriers hindering OAs' interaction with multiple mHealth device, including mobile application, smartphones, and wearable activity monitors (Baig, Gholamhosseini, Moqeem, Mirza, & Lindén, 2017; Foster & Sethares, 2014; Wildenbos, Peute, & Jaspers, 2018). All these inconsistent results, however, underlie the necessity for a more rigorously designed study to tease out the true relationship between technology fluency and device adherence.

Surprisingly, our findings also suggested no differences in both health‐related life quality and cardiovascular‐related health factors between users and nonusers, which might be due to the fact that individualized hypertension awareness plays a critical role on self‐care management practice (Badakhsh, Malek, Aghili, Ebrahim, & Khamseh, 2015; Zhang & Tan, 2019). On the one hand, individuals with already‐established healthy condition may desire a trigger of receiving positive reinforcement to maintain already‐established healthy lifestyle. On the other hand, OAs may feel it unnecessary to focus on hypertension self‐management in the case of no obvious symptoms over time. In addition, results of the current study indicate no significant difference on socioeconomic (SES), possibly due to the fact that the majority of participants had a monthly family income of <5,000 Yuan. This result is inconsistent with Peltzer and Phaswana‐Mafuya (2013), who pointed out that SES was significantly associated with the awareness of hypertension and strategies to engage with health system. Likewise, Yingling et al. (2017) has found that lower SES participants may be more likely to interact with a mHealth system than higher SES individuals. Nevertheless, more research is required to explore the path model connecting sociodemographic, quality of life and user adoption adherence among population with more diverse backgrounds.

Although the adoption of mHealth in OAs lags behind of younger individuals consistently (Searcy et al., 2019), a comprehensive understanding of the differences between device users and nonusers can identify barriers in device engagement and user adherence, thereby providing information to better allocate intervention resources in this population. As Burke et al. (2012) ranked “identifying strategies that improve self‐monitoring adherence” as an important factor in moving forward for mHealth‐based weight loss efforts. Our study was characterized by the assessment of user adoption of wearable BP monitoring device among OAs in a resource‐limited rural area. We observed that participants with higher levels of hypertension compliance also had high levels of device adherence. Thus, mHealth nonusers were more likely to be suboptimal hypertension compliance. The desire of OAs to engage the intervention is the main motivational inertia barrier, which is more difficult to overcome. Reaching these suboptimal participants may optimize mHealth tools in early intervention by healthcare setting to promote more engagement.

Limited technology usage and fluency should be noted to reduce potential disparities in the application of BP monitoring device to population‐based interventions in resourced‐limited settings. Our findings revealed that lower technology fluency was unlikely to impede integration of this mHealth BP monitoring system among OAs, which is consistent with the premise of the “new digital divide,” where technology may not be the most prominent along SES lines (Atienza & Patrick, 2011). This might be the variation in quality of data, sample sizes, and differing methodologies. In addition, our results suggested that a BP monitoring system incorporating a data collection hub may facilitate a population‐based BP control intervention, regardless of technology fluency and health‐related quality of life.

To date, rarely any studies have examined the adherence of mHealth BP monitor system among adults with age of or over 60 years. To the best of our knowledge, it is the first study to assess the associated influencing factors of mHealth technology adherence among OAs from a resource‐limited community, which is one of the strengths of our study. In addition, the present study also responds to the current calls for rigorous test of mHealth wearable device among populations with high risks of chronic diseases (Burke et al., 2015). However, several limitations must be acknowledged. To begin with, the cross‐sectional design of the study was unable to reflect the internal causal inferences, and thus, further research should be conducted to determine whether modification of these factors could lead to higher adherence of OAs. Furthermore, our findings were limited to a resource‐limited, rural settings where over half of participants reported unfinished primary school education, with <5,000 Yuan/month of family income. Future studies may extend to more diverse ethnic populations with various background and cohort studies to explore engagement factors of users. Finally, in this face‐to‐face interview‐based study, an overestimation of adherence was unavoidable due to social desirability, recall error, and other inherent misreporting. More advanced statistical methods should be adopted to adjust for control of confounding variables in the future.

## 5 CONCLUSION

Hypertensive elderly living in rural mountain areas are a vulnerable group in China. Moreover, the one‐child policy and internal migration from rural to urban areas have greatly increased “empty‐nested elders” (Zhang, Tan, et al., 2019; Zhang, Xu, et al., 2019; Zhang, Yang, et al., 2019). Thus, it is of high implication to study their adherence to wearable BP monitor device. In this study, the present outcomes revealed that OAs with higher levels of hypertension compliance were more likely to interact with a wearable BP monitoring device, whereas low technology, high HRQOL, and cardiovascular‐related health factors were not influencing factors of adherence to this mHealth BP monitoring system. The practical implications of these findings suggest that hypertension compliance may imply the requirement for additional interventions procedure targeting patients' attempts to control their BP.

## CONFLICT OF INTEREST

None declared.

## AUTHOR CONTRIBUTIONS

All authors reached final approval of the version to be published and declare that they have no conflicts of interest.

## Data Availability

The data that support the findings of this study are available from the corresponding author upon reasonable request.
